# Rental Housing Type and Self-Reported General Health and Mental Health Status: Evidence from the Canadian Housing Survey 2018–2019

**DOI:** 10.3390/ijerph21091181

**Published:** 2024-09-05

**Authors:** Shirmin Bintay Kader, Md Sabbir Ahmed, Kristen Desjarlais-deKlerk, Xavier Leloup, Laurence Simard, Catherine Leviten-Reid, Nazeem Muhajarine

**Affiliations:** 1 Department of Community Health & Epidemiology, College of Medicine, University of Saskatchewan, 107 Wiggins Road, Saskatoon, SK S7N 5E5, Canada; byp019@usask.ca (S.B.K.); sabbir.ahmed@usask.ca (M.S.A.); 2 Saskatchewan Population Health and Evaluation Research Unit (SPHERU), University of Saskatchewan, 107 Wiggins Road, Saskatoon, SK S7N 5E5, Canada; 3 Department of Business and Administration, University of Winnipeg, 515 Portage Avenue, Winnipeg, MB R3G 2E9, Canada; k.desjarlais-deklerk@uwinnipeg.ca; 4 Centre Urbanisation Culture Société, Institut National de la Recherche Scientifique, 385 Sherbrooke E, Montréal, QC H2X 1E3, Canada; xavier.leloup@inrs.ca (X.L.); laurence.simard@inrs.ca (L.S.); 5 Community Economic Development, Community-University Housing Research Laboratory, Cape Brenton University, 1250 Grand Lake Road, Sydney, NS B1M 1A2, Canada; catherine_leviten-reid@cbu.ca

**Keywords:** social-affordable housing, self-rated health, self-rated mental health, housing tenancy, National Housing Strategy (Canada)

## Abstract

Using the Canadian Housing Survey, 2018–2019, we examined self-reported general and mental health among tenants residing in various housing types, including cooperative, non-profit, government, and private housing. Adjusting for confounders, we discovered that tenants in not-for-profit housing reported the highest odds, over four and half times (odds ratio 4.63), of poor general health compared to tenants in privately owned housing in Canada. On the other hand, the odds were reversed for tenants in cooperative housing and government housing, with 24% and 33% lower odds of poor general health, respectively, compared to tenants in privately owned housing. Moreover, we found that tenants in not-for-profit (1.26) and government housing (1.43) reported higher odds of poor mental health. On the other hand, tenants in cooperative housing reported 42% lower odds of poor mental health than tenants in privately owned housing. Furthermore, we observed variations in the odds of poor general and poor mental health among tenants from different equity-seeking groups across different housing types. These findings highlight the importance of considering housing type and equity factors in understanding health outcomes among tenants.

## 1. Introduction

Housing is a basic human need [1] that directly links to an individual’s health [2]. The United Nations has long identified housing as a human right and that it should be affordable, safe, secure, culturally appropriate, accessible, and conveniently located [3]. Numerous studies have highlighted that housing conditions mitigate communicable and non-communicable diseases (NCDs) [4,5,6]. For example, poor housing quality can cause respiratory illness, cardiovascular disease, and physical, emotional, and general health problems [7,8,9,10]. Further, damp, humid, and extreme housing temperatures adversely affect respiratory health [9,11,12]. Previous authors argued that repairing physical structural issues in homes simultaneously improves residents’ health [12]. Homeownership affords physical and mental security, thus bolstering health [12]. Moreover, renters’ housing instability and inability to change dwelling structures eroded their health, particularly when compared to homeowners [11].

Studies also revealed that homeowners reported better mental health than private renters [13]. Moreover, tenants from various rental types reported significantly different odds of having NCDs, and these were attributed to the structural differences between houses or the neighbourhood infrastructure [14,15,16]. The literature suggests that different types of housing tenancy affect general physical and mental health differently. A population-based survey found that public housing residents were more likely to report poor health than other residents in the U.S. [17]. A study among tenants in South Wales mirrored these results. Tenants from randomly selected public housing rated their health as poor 2.5 times more often than did the general population in the same area [18]. Rabins et al. (1996) reported that older adults from public housing had a greater prevalence of psychiatric disorders than the general population of the same area [19]. A recent study from Manitoba, Canada, found that public housing residents were in poorer health than a matched counterparts and were more likely to use most types of healthcare services. The study also found that these same tenants did not change their healthcare use after moving in, with the exception of inpatient days in the hospital, which decreased compared to their matched cohort [20]. Moreover, Lubik and Kosatsky suggested that moving to cohousing (a housing type wherein residents participate in designing and managing their housing community) or cooperative housing improves the physical and mental health of its residents [21].

Delving deeper, qualitative studies have suggested that cooperative housing has both positive and negative impacts on self-rated physical, mental, and general health. A qualitative study of 26 participants from eleven separate cooperative housing units expressed mixed feelings of emotional and physical well-being [22]. This study focused on communal living, a living arrangement that purportedly fostered a positive sense of community among participants. The study reported, paradoxically, that this particular type of living arrangement exerted adverse effects on residents’ emotional and physical well-being, which is attributed to the challenges posed by shared spaces [22]. Participants in this study also reported mixed feelings about the manner in which the cooperative housing was governed and its involvement in decision-making. The authors observed that effects such as stress, fatigue, guilt, and insecurity were due to conflict between the residents and governance. On the other hand, the same study also reported that the residents felt confident that their expectations were met by the governance body [22]. Houle et al. (2018) also identified a mixed impact of perceptions of the residential environment on the well-being of the residents in a study of public housing tenants from Montreal [23].

Despite the well-documented health impacts of inadequate housing, and the recognition that housing is a fundamental human right, Canada has failed to ensure adequate housing for all its residents [24]. In 2017, the Canadian Mortgage and Housing Corporation (CMHC), on behalf of the Canadian government, established the National Housing Strategy (NHS) to serve as a unified regulatory framework for the country [25]. The NHS seeks to provide Canadians with a place to call home and to reduce national homelessness. The NHS, to some degree, has prioritised community housing sectors by collaborating with non-profit and cooperative housing providers and has developed a comprehensive plan that focuses on vulnerable Canadians, including women, children, Indigenous people, seniors, and individuals with disabilities, as well as those suffering from mental health and addiction issues [25]. While housing policy is complex, and the objectives of these policies have changed over time, the current strategic plan of the NHS is to increase the accessibility of affordable houses to meet the needs of Canadians, thus reducing homelessness [26]. As per CMHC, in Canada, affordable housing is defined as those houses whose costs are less than 30% of a household’s before-tax income [27]. Providers of affordable housing, however, could include the government, cooperatives or non-profit organizations (public or private), and affordable housing can be either owned or rented [26,28,29].

Government-owned and -operated housing, generally known as public housing, is made available to low-income earners and charges lower rent, typically set at 30% of income. Most government housing in Canada was built between 1949 and 1978, and tenants receive housing based on (often long) wait lists [30]. Traditional target groups for this housing are families and seniors, with this housing type also more recently prioritising other groups, including people who are at risk of homelessness. Cooperative housing is generally organised on a not-for-profit basis in Canada, and a proportion of these housing units are subsidised and assigned to low-income tenants [21]. Some cooperatives have, thus, a mix of higher and lower-income residents, while others are organised for specific populations, including low-income families or people with disabilities [21,31]. In addition, faith-based organizations, community development corporations and other types of community-based organizations sponsor affordable housing known as not-for-profit housing. These units are developed by these organizations to respond to specific housing needs of vulnerable groups, for example, older tenants, those who struggle with mental health and/or substance use [32]. The rent for not-for-profit housing is usually set below the market rate. Market housing refers to that which is privately owned and may be available for rent at a market rate. That said, if the tenants pay more than 30% of their income towards rent, they may be eligible to apply for additional financial assistance to afford the rent. It is important to note that subsidies, such as housing allowances and rent supplements, are not made available to all those in need of such assistance [26].

Unaffordable housing can have a detrimental effect on self-rated general and mental health [33]. Several factors contribute to this negative health outcome, including rent burden, socio-demographic disparities among tenants, and harmful coping strategies such as living in overcrowded or doubled-up households [34]. While specific evidence for Canadian renters is lacking, significant disparities are observed among US renters in affordable housing, primarily based on income, ethnicity, and race [33,35]. This suggests that similar patterns might be present in Canada, making the US data relevant for our context. Due to the increasing burden of unaffordable housing in Canada, it is critically important to assess the health status of renters living in different housing types [36]. Our goals in this report are in line with research funds provided through the National Housing Strategy to better understand housing for those in greatest need [26]. To complement the National Housing Strategy, the Canadian Housing Survey (CHS) was launched in 2018 and conducted over 2018–2019. This biennial national survey aims to understand the housing needs and experiences of Canadians, particularly those in vulnerable priority groups. Using CHS cycle 2018–2019 data, we investigated the associations between different housing types and renters’ general health and mental health.

We know from previous investigations that the results of associations between the physical, mental, and general health of residents and different housing types are mixed [17,18,20,22]. Our project focused on individuals with the greatest need for housing, aiming to identify the differences in health status among tenants. While homeowners, as well, might have distinct health relationships based on their housing needs, comparative studies have consistently shown that tenants report poorer health than homeowners. Therefore, the current study aims to understand how renters who reside in tenancy housing types classified by providers, specifically, government, not-for-profit, cooperative, and privately owned housing in Canada, describe their general health and mental health. We know that tenants’ individual and social characteristics, such as age, gender, household income level, ethnocultural minority status, and household composition, are critical determinants that pattern tenancy housing type and health relationships. To identify the moderating effect of these factors, we investigated whether tenancy housing types and self-rated general health and mental health associations vary across different equity-seeking groups. Additionally, this study incorporates a variable on subsidies received, providing an overview of the moderating effects of subsidies on housing type and health. However, due to the cross-sectional nature of the data, this study does not examine the impact of living in units with different provider types or receiving subsidies on general health or mental health. Instead, it focuses on identifying general health and mental health differences among residents of various tenancy housing types in Canada.

## 2. Methods

### 2.1. Data Source

We used secondary data from the first round of the Canadian Housing Survey (CHS) 2018–2019. This nationwide cross-sectional survey, implemented by Statistics Canada, and sponsored by CMHC, covered all ten provinces and three territories of Canada. Data were collected from eligible adults using online surveys for provinces and in-person surveys for territories between 1 November 2018, and 31 March 2019. Both homeowners and tenants who participated in this survey provided information about demographics, housing needs, life experiences, health status, neighbourhood, etc. In each household, the individual responsible for housing decisions (referred to as the reference participant) was asked to fill out the survey questionnaire. The CHS sample excluded residents of institutions, members of the Canadian Forces living on military bases, and people living in First Nations communities (i.e., on-reserve) or other Indigenous settlements. A stratified sampling method was followed based on each province’s Census Metropolitan Areas (CMAs). More than 61 thousand households (61,764) participated in the survey, with a response rate of 50%. Household members aged 15 years or older were asked to participate in this study, without restriction at the highest age limit. All responses were collected from the participants directly, and a proxy response was accepted for questions about other household members only. For our analysis, we considered data from respondents who indicated that they were not homeowners but were renters of the homes they lived in. This included data from 26,371 renters. Any missing data either at the personal level or the household level were imputed. Detailed information about this survey, including the methodology, questionnaire, etc., can be found elsewhere [37].

### 2.2. Variables

For the outcome variables, self-reported general health and mental health status were the two outcome variables for this study. These are well-known, valid, single, self-administered, and globally used health measures that reflect an individual’s current state of health, both general and mental health [38,39,40,41,42]. The participants were asked, “In general, how is your health?” and “In general, how is your mental health?” to assess their general health and mental health status, respectively. Both questions’ responses were categorised as excellent, very good, good, fair, and poor. For our analyses, we have re-categorised the responses as good (combining excellent, very good, and good) or poor (combining fair and poor), as has been conducted in previously published articles [43,44].

For the exposure variables, the type of tenancy house based on the landlord and whether they received a subsidy were the two key exposure variables for this study. Participants were asked, “Who is your landlord?” and “Is the rent for your dwelling subsidized?” to assess these variables, respectively. In the original survey, the response for tenancy types based on who owns the housing (landlord) was grouped into four categories, including government, not-for-profit, cooperative, and privately owned. Receipt of subsidies was recorded as a dichotomised response, yes or no.

For the co-variables, covariates were selected a priori based on their prior established relationships (i.e., the literature and/or theorised relationships) with general health or mental health outcomes. A list of all covariates and their descriptions used in the analysis are presented in Table 1. Among the covariates, we identified equity-seeking groups as people facing significant barriers to rental housing (with or without subsidy) due to various factors, such as age, gender, ethnocultural identity, household composition, and household income. These variables pertaining to equity-seeking groups were used as moderators in the associations between tenancy housing type and self-rated general health and mental health.

### 2.3. Statistical Analysis

Before commencing the formal analyses, we prepared a master data file by merging household and individual data sets, and then, we filtered renters’ data (our study sample). Then, we edited, coded, and recoded our study variables. Due to the low proportion of missing cases (6.2%), we conducted a complete case analysis. Both descriptive and inferential statistical methods were used in this analysis. Descriptive statistics were used to summarise the study variables, and cell counts were checked to comply with Statistics Canada reporting guidelines. Bivariate analyses (chi-square tests) were conducted to determine the association between the socio-demographic, household, and neighbourhood characteristics of the tenants and the general health and mental health status. The adjusted associations between rental housing type and poor general health and mental health status were estimated by multivariable binary logistic regression models (see Supplementary Table S2). Final regression models were estimated adjusting for covariates and interaction terms in the models. The variables for assessing interactions were selected from the equity-seeking group [45] (namely subsidised housing, age, gender, ethnocultural identity, household composition, household income, and neighbourhood safety); we hypothesised that different types of marginalities pertain to differential associations between rental housing type and general health and mental health [45]. Only significant interaction terms were included in the final regression models. The regression results were expressed as odds ratios (ORs) with 95% confidence intervals (CIs), and the receiver–operator curves were used to determine the model fitness (see Supplementary Figure S1). The variance inflation factor (VIF) was used to check multicollinearity among the independent variables. All statistical analyses were performed using Stata SE v.15 [46], and *p* < 0.05 was considered a significant result. All survey estimates were weighted to ensure sample representativeness [46].

## 3. Results

### 3.1. Sample Characteristics

Table 2 shows the weighted percentage of sample characteristics and the distribution of the respondents based on sociodemographic and housing-related factors. Most of the tenants resided in private housing (83.6%) and lower proportions in cooperative (6.8%), not-for-profit (3.1%), and government (6.5%) housing. Most tenants (85.7%) indicated that they do not receive subsidies independent of the housing type. The study participants were diverse in age, with the highest percentage in the 25–34 years group (41%). Gender distribution was nearly balanced, with women representing 51.7% of the sample. More than two-thirds (71.9%) of the respondents were white, and 28.1% identified as BIPOC (Black, Indigenous, and People of Colour). The respondents had diverse distributions across educational status, occupation, and family size groups. The most common education level was a high school diploma (23.7%), and 54.6% reported being employed at the time of data collection. Small households or family sizes were common (43.4% lived alone and 29.9% consisted of two people). About half (51.6%) of the respondents were not in census families. Only 36.2% of the respondents had household incomes above 120% of the median income of the province of their residence. Many respondents were from Ontario (36.7%), followed by Quebec (30%). In terms of housing factors, 47.7% reported housing satisfaction, and 64.2% reported no dwelling issues. Residential mobility shows that one-third (33.6%) of tenants resided in their current location for 2–5 years. Most felt very safe (25.9%) and were satisfied (33.4%) with their neighbourhood.

The distribution of the weighted percentage of the sociodemographic characteristics of the study participants across the different types of housing (government, not-for-profit, cooperative, and privately owned housing) is shown in Supplementary Table S1. Most of the respondents were in privately owned housing; the lowest percentage of renters were in not-for-profit housing (Supplementary Table S1).

Considering the sociodemographic profile of tenants in each of the four types of housing, the tenants in government housing tended to be older, with 11.6% aged 65 and older compared to 2.8% in the 25–34 years group. A similar distribution was also seen for tenants in not-for-profit housing (5.9% in 65+ years vs. 1% in 25–34 years). In contrast, privately owned housing is occupied by younger tenants, with 91.3% in the 25–34 age group, and 74.8% in the 65 and older age group. In contrast, privately owned housing is occupied by younger tenants, with 91.3% in the 25–34 age group, and the least reported belongs to the 65 and older age group (74.8%). In contrast, privately owned housing is occupied by younger tenants, with 91.3% in the 25–34 age group, and 74.8% in the 65 and older age group. The male-female ratio was nearly equal for privately owned and not-for-profit housing. However, in government and cooperative housing, the prevalence of female tenants was higher than that of male tenants (male:female—5.0:7.9 and 5.4:8.1 for government and cooperative housing, respectively). BIPOC groups were more prevalent in government and cooperative housing than their white counterparts (7.8% vs. 6.0%, and 9.8% vs. 5.6%, respectively). Interestingly, with increasing income, the percentage of tenants in privately owned housing increased (70.40% to 91.90%), while the percentage of tenants in other housing types decreased. Tenants with a household income below 40% of the median income in government housing, not-for-profit housing, and cooperative housing were 14.6%, 6.1%, and 9%, respectively. On the other end, tenants with a household income that was more than 120% of the median were only 1.9%, 1.5%, and 4.8% in government housing, not-for-profit housing, and cooperative housing, respectively (Supplementary Table S1).

### 3.2. Health Disparities across the Sociodemographic Factors

Overall, 19.5% and 16.3% of the respondents reported poor general and mental health, respectively. Table 2 shows that, among the oldest age group (65+ yrs.), 26.2% reported having poor general health. On the contrary, the highest prevalence of poor mental health (19.3%) was found in the youngest age group. The prevalence of poor general and mental health among women was 20.0% and 17.5%, respectively. Among those identified as BIPOC, 17.4% and 13.6% reported poor general and mental health, respectively (Table 2). Among the participants with less than a high school education, 33.1% and 19.1% reported poor general and mental health, respectively (Table 2). The prevalence of poor general health (72.7%) and mental health (50.7%) was also predominant in the long-term disabled group. Among the respondents with a small household or family size (one person), the prevalence of poor general health (23.9%) and mental health (18.4%) was higher than the respondents who had more family members. Among people not in census families, 22.6% had poor general health and 18.8% had poor mental health. Respondents from households with an income below 40% of the median household income of their province reported 36.3% and 32.6% poor general health and mental health, respectively. The highest prevalence of poor general health among renting households was reported in Nova Scotia (23.6%) and New Brunswick (23.9%), whereas the highest poor mental health was reported in the Yukon (26.1%) (Table 2). The disparities across sociodemographic factors significantly (*p* < 0.001) differed between poor and good in both general health and mental health outcomes.

### 3.3. Health Disparities across Housing-Related Factors

The highest prevalence of poor general health (40.8%) and poor mental health (31.4%) was reported by participants who were very dissatisfied with their dwellings. Similarly, a higher prevalence of poor general health (33.8%) and poor mental health (28.7%) was reported by respondents having more than two problematic dwelling issues. The prevalence of poor general health (24.2%) and poor mental health (22.7%) was higher for respondents whose homes needed any repairs. Regarding residential mobility, tenants who had resided in their homes for the last ten years or more reported a higher prevalence of poor general health (27.0%). On the other hand, tenants who moved within less than two years prior to the survey reported the highest prevalence of poor mental health (17.2%). Among respondents who were dissatisfied with their neighbourhood, 33.9% reported poor general health and 38.0% reported poor mental health. Similar to other housing factors, tenants who felt very unsafe in their neighbourhood reported a higher prevalence of poor general health (34.6%) and poor mental health (30.6%) than the other groups (Table 2). Like sociodemographic factors, the disparities across the housing factors also significantly (*p* < 0.001) differed between poor and good for both general health and mental health.

### 3.4. Association between Housing Tenancy Types and Tenant’s Poor General Health and Mental Health

Table 3 shows that the prevalence of poor general health (37.0%) and poor mental health (22.7%) was highest among government and not-for-profit housing tenants, respectively. After adjusting for sociodemographic and housing factors, we found that tenants from not-for-profit housing reported more than four times higher adjusted odds for poor general health (AOR = 4.63; 95% CI: 4.15–5.16), and tenants in government housing reported 43% higher odds for poor mental health (AOR = 1.43; 95% CI: 1.34–1.52) in comparison to those tenants in private housing (Table 3). Moreover, those tenants who received subsidies had 21% higher odds of poor general health (AOR = 1.21; 95% CI: 1.20–1.22) and 5% greater odds of poor mental health (AOR = 1.05; 95% CI: 1.03–1.06) compared to those who had not received any subsidy.

### 3.5. The Association between Tenancy Housing Type and Health Status Moderated by Equity-Seeking Groups

#### 3.5.1. General Health

**Figure 1:**
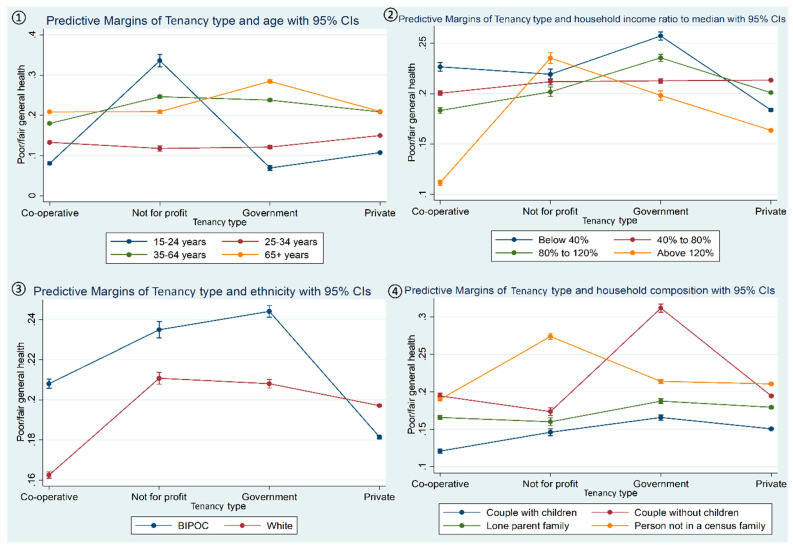
Interaction plots showing the moderating roles of (**①**) age, (**②**) household income ratio to provincial median, (**③**) ethnicity, and (**④**) household composition in the association between tenancy type and poor general health status.

The Figure 1 cluster shows significant interaction plots that reveal how equity-seeking groups, namely, ethnocultural identity (BIPOC vs. White), household income level, and household composition, have moderated the relationship between tenancy type and poor general health. Figure 1(①) presents the interaction plot showing the association between tenancy type (cooperative, not for profit, government, privately owned) and poor general health status affecting age groups differently. The highest probability of poor general health is seen among the youngest tenants in not-for-profit housing. Figure 1(②) shows the associations between tenancy type on poor general health differentially affecting household income levels. The expected pattern of the lower the household income the greater the probability of poor general health is interrupted by tenants with a household income at least above 120% of the provincial median, showing a wide fluctuation. For this higher household income group, the probability of poor general health is the lowest for tenants in cooperative housing and markedly higher for tenants in not-for-profit housing. Figure 1(③) reveals that BIPOC tenants, compared to Whites, had a higher probability of poor general health, especially in government and not-for-profit housing. Figure 1(④) shows the probability of poor general health being affected by different family or household compositions. Across the four family/household types, a higher probability of poor general health was seen among tenants without children in government housing, as well as in tenants living with people who they are not related to in not-for-profit housing. It is notable that, of all household types, couples with children who lived in cooperative housing had the lowest probability of poor general health across all housing tenancy types.

#### 3.5.2. Mental Health

**Figure 2:**
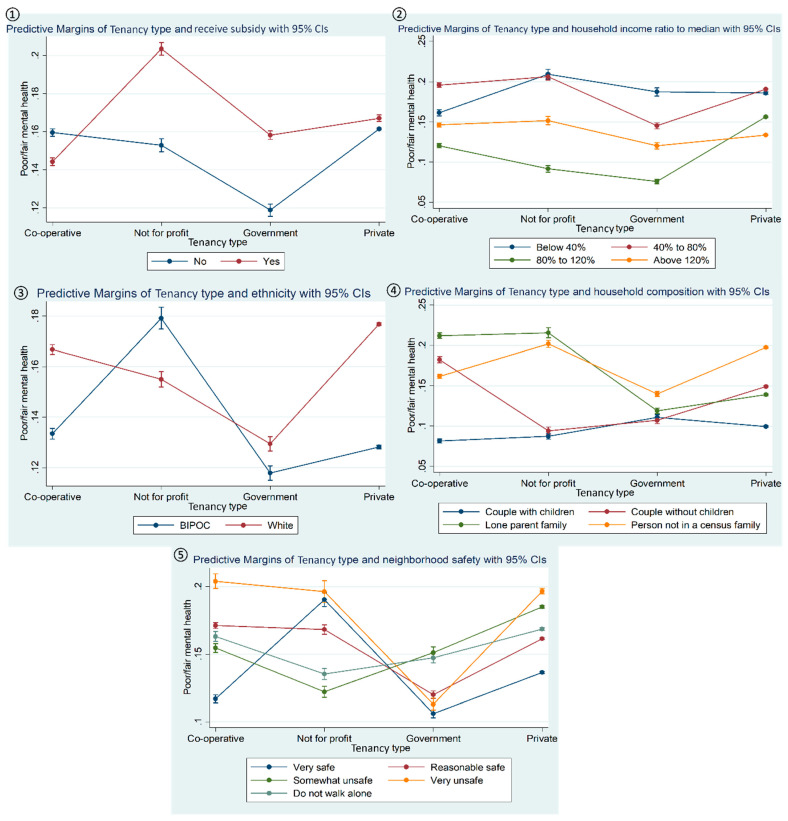
Interaction plots showing the moderating role of (**①**) receiving housing subsidies, (**②**) household income ratio to provincial median, (**③**) ethnicity, (**④**) household composition, and (**⑤**) neighbourhood safety in the association between tenancy type and poor mental health status.

The Figure 2 cluster shows the interaction plots for equity-seeking groups, e.g., ethnicity, household income, and those who received a subsidy in relation to tenancy type and poor mental health. status. Figure 2(①) shows that the probability of poor mental health was higher among the tenants in not-for-profit housing who received subsidies. Moreover, the tenants who stated that they had not received housing subsidies and resided in government housing had a lower probability of poor mental health. Figure 2(②) shows tenants in the two lowest household income groups (at least 80% below the provincial median income) reported the highest probability of poor mental health among all groups, especially for those in not-for-profit housing. Figure 2(③) shows that, for BIPOC tenants, compared to White, the probability of poor mental health was high for those in not-for-profit housing. In contrast, BIPOC tenants in government housing had the lowest probability of poor mental health. Figure 2(④) shows the moderation effects of family/household compositions on the association between tenancy type and poor mental health. The highest probability of poor mental health was among lone-parent families who were residing in either cooperative or not-for-profit housing. In contrast, couples with children living in cooperative housing reported the lowest probability of poor mental health. Figure 2(⑤) shows tenants in cooperative housing who indicated that their neighbourhoods were unsafe reported a higher probability of poor mental health.

## 4. Discussion

Tapping into the largest population-wide survey on housing in Canada to date, this study examined both the general health and mental health status among tenants living in the different types of rental housing in Canada, namely those that are owned or sponsored by the government, not-for-profit entities, cooperative entities, or private landlords. It revealed a few important insights. First, one in five tenants (19.5%) reported poor general health and slightly over one in six tenants (16.3%) reported poor mental health in this sample. Importantly, focusing on general health, the results showed that tenants from not-for-profit housing reported the highest odds, over four and half times (odds ratio 4.63), of poor general health compared to tenants in privately owned housing. For tenants in cooperative housing and in government housing, the odds were reversed, with 24% and 33% less likelihood of poor general health, respectively, compared to tenants in privately owned housing. These estimates were reached after statistically controlling for many covariables representing alternate explanations.

Given the cross-sectional design of the survey, these findings raise the question of whether they could have been observed due to the self-selection of people who already have poor health into different types of tenancy housing. On the one hand, the patterns of systematically different higher odds of poor general health observed in specific subgroups of tenants in this study—for example, younger respondents, those with higher household income, and white respondents—could be considered unlikely and therefore inconsistent with this ‘reverse causation’ hypothesis. It is also noteworthy that study participants in the youngest age category had the lowest adjusted odds of general health (see Supplement Table S2). At the same time, however, it is important to acknowledge how different housing types have been developed in Canada, especially for those in greatest need in different regions across the country, with not-for-profit and government housing providers, in particular, playing unique roles in providing housing to those not well served through private landlords. One example is not-for-profit organizations specifically developed with the mandate to provide housing to youth-in-need who have experienced, or are at risk of experiencing, homelessness, or recovering from substance use. Furthermore, younger renters in not-for-profit housing may have specific health needs tied to the eligibility criteria, such as ongoing substance use recovery or needing disability accommodations. In this study, only 1.3% were in the youngest age group, 15–34, in not-for-profit housing, compared to 90.2% in the same age group in privately owned housing. Given the targeted support that these programs provide, the tenants’ health may likely be more acute or complex. Self-selection, especially for youth in this case, is a second possible interpretation of these findings, which merits further exploration using a different study design.

Second, this study reveals that poor general health across tenancy housing is experienced and reported differently for younger vs. older, people with lower vs. higher household income, BIPOC (Black, Indigenous, and Person of Colour) vs. White, and for those with children vs. those without children (household composition). The youngest of the tenants, 15–24 years of age, had the highest probability of all age groups reporting poor general health associated with living in not-for-profit housing. While the effect of income moderated the probability of poor general health, as expected—that is, the lower the household income, the greater the probability of poor general health across tenancy types—this pattern was interrupted by tenants with the highest household income in the sample (i.e., income at or above 120% of provincial median). For the higher income group, the probability of poor general health was highest in not-for-profit housing tenancy. For tenants who are BIPOC, the probability of poor general health was consistently more significant than for white tenants across tenancy housing types, with one exception. BIPOC tenants in privately owned housing had a lower probability of poor general health compared to their White counterparts.

The third insight revealed in this study is that the two health outcomes, general health and mental health, associated with the tenancy housing types showed similar yet different results, buttressing the argument that while similarities exist, the mechanisms producing these two health status outcomes are different. The adjusted odds of poor mental health were higher for tenants living in not-for-profit (26 percent higher) and government-owned housing (43 percent higher), compared to tenants in privately owned housing. In contrast, for tenants in cooperative housing, the odds of poor mental health were 48 percent lower.

In terms of consistency, as mentioned, our study found that tenants in government housing had lower odds of poor general health than tenants in privately owned housing. On the other hand, tenants in not-for-profit housing had higher odds of poor general health, which is somewhat consistent with other studies [17,18,47]. Digenis-Bury et al. (2008), for example, found similar odds for tenants in public housing, with 4.58 times higher odds of poor overall health than the other residents in Boston City. Wiggers et al. (2001) reported that tenants from public housing are 2.5 times more likely to report poor health than the general population from South Wales. Tomioka et al. (2019) reported that tenants from publicly subsidised housing had 1.23 times higher odds of poor self-rated health than homeowners in Japan.

Beyond examining poor general health, this study delved into the mental health status of tenants from different tenancy housing types. While no directly comparable nationwide study corroborates our specific findings, existing research, such as Simning et al. (2011), offers similar outcomes. Simning et al. reported 80% elevated odds of anxiety disorders and 40% increased odds of mood disorders observed among public housing tenants in comparison to their non-public housing counterparts [48]. In this study, we report similar higher odds of poor mental health for tenants in not-for-profit and government housing in comparison to tenants in privately owned housing, which is a clearer comparison than what most previous studies had employed.

In addition to different types of housing tenancy, we found tenants who received subsidies reported poor general health and poor mental health independent of tenancy type. Jenkins Morales and Robert (2024) conducted a comparative follow-up study between older renters receiving subsidies and those not receiving housing subsidies. They found that older renters who received subsidies reported worse self-rated health compared to renters with no subsidies at the baseline [49]. However, after two years of follow-up, they found no significant difference in health for subsidised vs. unsubsidised renters. Jenkins Morales and Robert (2024) discussed that the possibility of prioritising those with poor health at entry to receive subsidies might be the possible cause of initial higher poor self-rated health.

This consistent pattern across studies from different jurisdictions (e.g., Freund et al., 2023 [50]; Tomioka et al., 2019 [47]; Wiggers et al., 2001 [18]) of reporting poor self-rated health among social and affordable housing tenants could be explained by the housing and neighbourhood quality. Public housing often incorporates a design where numerous individuals inhabit a compact geographic space, facilitating streamlined service delivery [51]. Thomson et al. demonstrated how constrained space and crowded living conditions directly impact overall health, an association also evident in our study population (see Supplementary Table S2) [52]. On the other hand, Palacios et al. (2021) identified that residents in housing that is in need of repair reported poor physical and mental health, which is a finding similar to ours (see supplementary Table S2) [53]. Furthermore, sometimes, the unfavourable neighbourhood conditions in which social housing is located restrict access to critical services, such as healthcare and healthy food options, contributing to poor health outcomes [54]. Moreover, studies suggested that housing insecurity, continuously living in social housing, and multiple transitions can cause worse mental health, which indicates mechanisms through which rental status affects the mental health of people [55,56].

Although multiple studies established the association between different types of public housing and general health [18,47,50], none, to our knowledge, have reported the association between housing types and general health based on different age groups. A study reported that Canadian children and youth from crowded housing and unaffordable housing reported several illnesses, including malnutrition and psychological distress [57]. Also, Baker et al. reported younger individuals, similar to the age group in our study (15–22 years), who lived in poor-quality dwellings reported higher odds of poor general health, which indirectly supports our study findings [10]. In contrast, older adults (65 years and older) who are tenants in government housing were the second-highest group who reported higher odds of poor general health. These diverse results across age groups and housing types may well reflect the neighbourhood environment of government housing, which was previously identified as an adverse factor in developing poor physical health by Tomioka et al. [47].

Coming back to the results of the moderation analysis, as expected, based on the body of previous studies [58,59], those tenants in equity-seeking groups, namely ethnic minorities (BIPOC), had a greater probability of poor health (general health and mental health) associated with the not-for-profit and government housing tenancy types. As mentioned, this may in part be due to the poor quality of the housing and, by extension, the poor neighbourhood quality in which these types of tenancy housing may be located. In addition to the lack of amenities (parks and recreational), assets (bike and walking paths), and helpful services (grocery stores and libraries) in the surroundings of these tenancy housing, there are perceptions of disorder and lack of safety (see Figure 2(⑤)) that could further set back the equity-seeking residents of these neighbourhoods [60,61]. Additionally, residing in deprived neighbourhoods due to living in social and affordable housing exacerbates the impact of poor health outcomes due to a lack of social relations for ethnic minority groups [60].

Furthermore, we found that, for both health statuses, couples with children who reside in cooperative housing reported the lowest likelihood of experiencing poor general and poor mental health. One explanation might be that families with children, compared to those who are living alone, may experience less loneliness and enhanced social support, leading to better general and mental health. Hansen et al. (2021) reported that people who lived alone in their houses had twice the odds of getting poor mental health than those who lived with children [62]. A previous study found that Danish individuals experienced loneliness due to living in deprived neighbourhoods, leading to isolation and an increased likelihood of engaging in risky behaviours, such as low vegetable or fruit intake, less physical activity, increased alcohol consumption, and smoking, which might be a potential reason behind poor health outcomes among lonely parents in our study [63].

This psychosocial element is illustrated well by a few of the findings that were unexpected in the moderation analysis. Specifically, the tenants who were 15–24 years old and the tenants with household income greater than 120% when compared to the provincial median indicated a greater probability of poor health when they live in not-for-profit or government housing, and BIPOC tenants indicated a lesser probability of poor health when they are tenants in privately-owned housing. These results indicate the complicated nature of the provision of affordable social housing in Canada presently. The provision of social housing alone is not sufficient to enhance the health and well-being of tenants residing in them; other things (i.e., better amenities and services accessible in the neighbourhood, perceived or real neighbourhood safety) need to be provided or enhanced. There is a need to monitor the quality of not-for-profit and government tenancy housing and to destigmatise and enhance the reputation of these types of housing as one among an array of housing choices available for Canadians.

Leviten-Reid et al. (2022) found that 47% of the public housing in a Nova Scotian municipality in Canada was in highly deprived (material and social) areas. They argued that the provincial and federal governments needed to assess the neighbourhoods further when they are developing new affordable housing. They emphasise the importance of conducting a comprehensive socio-economic assessment of the residents before implementing any housing policy [64]. While proximity to amenities has been evaluated in Canada by CMHC, so should material and social deprivation issues be addressed, along with housing needs to improve health.

## 5. Strengths and Limitations

This study has several strengths. We report the association between specific tenancy housing types (government, not-for-profit, cooperative, and privately owned) on general health and mental health. Previous studies have only reported the general health or mental health of tenants from only one or two different social and affordable housing types [17,47,56,65]. Further, we have considered the moderation effect of equity-seeking groups, which allows us to find out how differently the tenants suffered. Previous reports have underplayed equity considerations [66], especially when using nationally representative data. This paper closes that gap. Our nationally representative data from the CHS allowed us to have sufficient statistical power and a large enough sample size to delve into the moderating effect of social and affordable housing types on equity-seeking groups. Despite this large sample size, we are unable to establish causal relations between housing and health due to the cross-sectional nature of the study design. However, we have contended that the specific types of associations we have observed are not consistent with solely a self-selection (to specific tenancy housing types) or reversed-cause hypothesis. We relied on self-reported general and mental health status for health outcomes, which can introduce potential subjective bias. However, self-reported general health is one of the most widely studied health-status variables, which has been shown to closely correlate with clinical outcomes, including hospitalisations and visits to physicians [67,68,69]. We have not captured the neighbourhood environment in detail, which might be a group-specific confounder in the relationship between different socioeconomic and BIPOC groups with health outcomes. This paper is among the first we have written using the first round of the Canadian Housing Survey, despite having limited access to variables regarding housing affordability. The primary focus was identifying general health and mental health status differences among tenants from various housing situations. The findings from this study lay the groundwork for future research.

## 6. Conclusions

This study provides valuable insights into the need for targeted interventions to address health disparities among tenants in various social and affordable housing types. Policymakers should prioritise initiatives aimed at improving the general and mental health of individuals residing in not-for-profit and government housing, particularly focusing on younger adults, low-income tenants and BIPOC communities. For example, supportive housing programs that combine affordable housing with access to healthcare and social services have been effective in addressing the needs of individuals with chronic health conditions or those in recovery from substance use. Additionally, policymakers should ensure dedicated funding for community-based programming, such as ones related to food access, community development, and recreational opportunities. Moreover, health-enhancing interventions, such as better mental health support integrated into housing and social support in not-for-profit housing, can play a crucial role in improving overall well-being [70].

Efforts should be directed towards enhancing mental health resources for government housing tenants, acknowledging the elevated risk identified in this study. The results underscore the importance of intersectional approaches in housing policies, recognising the unique challenges faced by different demographic groups, which indicates that more subgroup analysis and longitudinal study can shed a causal relation on specified vulnerable groups. Furthermore, this study utilised data from the first wave of the National Housing Survey conducted across Canada. These data provided primary evidence on the health status of renters in different types of housing, serving as a foundation for future researchers to develop new research hypotheses. A longitudinal analysis is needed to rule out the influence of neighbourhood factors, housing conditions, and pre-existing health conditions on the health of renters.

## Figures and Tables

**Table 1:** Variables and their descriptions and categories used in this study.

Variable	Description and Categories
Age	Age (in years) of the survey respondents, categorised as: 15–24 years, 25–34 years, 35–64 years, and 65+ years.
Gender	Self-reported gender identity, categorised as Male, and Female.
Ethnocultural identity	Ethnocultural background, categorised as Black, Indigenous, and People of Colour (BIPOC), and White.
Educational status	Highest educational level, categorised as Less than high school, High school diploma, Trade certificate/Diploma, College/non-university degree, University certificate/diploma, Bachelor, and University degree/diploma above Bachelor
Occupation	Main activity in past 12 months, categorised as Working, Looking for work, Going to school, Keeping house, Caring for other family members and young children, Retired, Long-term illness/disability, Doing volunteer work, and No main activity.
Household or Family size	Total no. of family members categorised as 1, 2, 3, 4, and 5+.
Household composition	Household composition was categorised as Couple with children, Couple without children, Lone parent family, and Person not in a census family.
Dwelling satisfaction	Dwelling satisfaction among the tenants was categorised as Very satisfied, Satisfied, Neither satisfied nor dissatisfied, Very dissatisfied.
Dwelling issues	Three variables represented housing quality included dwelling issues—presence of mould/mildew, presence of unwanted pests, and presence of poor air quality. Based on these variables we have created a composite variable and categorised as No issue, One issue, Two issues, and Three issues.
Dwelling needs repair	Dwelling needs repair was dichotomised as No and Yes.
Residential mobility	Length of stay at current residence, categorised as <2 years, 2–5 years, 6–9 years, and 10+ years.
Neighbourhood satisfaction	Neighbourhood satisfaction was categorised as Very satisfied, Satisfied, Neither satisfied nor dissatisfied, Dissatisfied, Very dissatisfied.
Neighbourhood safety	Neighbourhood safety was categorised as Very safe, Reasonably safe, somewhat unsafe, Very unsafe, and Do not walk alone.
Household income ratio to median	Annual household income was compared with the provincial median, which takes account of income variations between provinces and territories in relation to the respective size of their economy. Household income indicator was based on the adjusted income of households according to their size (adjusted household income was obtained by dividing total income by the square root of their size). Household income ratio to provincial median income was categorised as Below 40%, 40% to 80%, 81% to 120%, and Above 120%.
Province of residence	Province/territories of current residence categorised as Newfoundland and Labrador, Prince Edward Island, Nova Scotia, New Brunswick, Quebec, Ontario, Manitoba, Saskatchewan, Alberta, British Columbia, Yukon, Northwest Territories, and Nunavut.

**Table 2:** Background characteristics of the tenants, prevalence of self-reported poor general health and poor mental health status, Canadian Housing Survey 2018–2019 (N = 26,371).

Variables	Weighted %	General Health Status (Poor) Weighted %	Mental Health Status (Poor) Weighted %
	N = 26,371 (100%)	n = 5143 (19.5%)	n = 4299 (16.3%)
Tenancy housing type			
Government	6.5	37.0	20.3
Not for profit	3.1	30.8	22.7
Cooperative	6.8	22.4	17.4
Private	83.6	17.5	15.7
Received Subsidy			
No	85.7	16.8	15.2
Yes	14.3	35.8	23.2
Age (in years)			
15–24	6.0	7.5	19.3
25–34	41.0	9.8	17.1
35–64	30.4	22.3	18.5
65+	22.6	26.2	9.9
Gender			
Male	48.3	18.9	15.1
Female	51.7	20.0	17.5
Ethnicity			
BIPOC	28.1	17.4	13.6
White	71.9	20.3	17.4
Educational status			
Less than high school	13.7	33.1	19.1
High school diploma	23.7	22.8	18.0
Trade certificate/diploma	8.2	20.7	17.0
College/non-university degree	19.0	19.9	16.6
University certificate/diploma	5.2	15.2	16.7
Bachelor	18.2	11.4	14.4
University degree/diploma above Bachelor	12.0	10.1	11.6
Occupation			
Working	54.6	11.3	13.6
Looking for work	3.0	15.8	21.6
Going to school	7.1	9.3	15.1
Keeping house	2.6	24.4	16.8
Caring for other family members and young children	3.9	21.0	18.8
Retired	18.1	25.1	9.0
Long-term illness/disability	7.0	72.7	50.7
Doing volunteer work	1.2	19.2	17.5
No main activity	2.6	33.3	24.2
Household or Family size			
1	43.4	23.9	18.4
2	29.9	15.6	15.5
3	10.6	16.5	16.8
4	8.7	16.7	11.9
5+	7.3	17.0	11.9
Household composition			
Couple with children	17.8	14.6	10.0
Couple without children	19.6	14.9	13.0
Lone parent family	11.0	21.2	20.7
Person not in a census family	51.6	22.6	18.8
Household income ratio to median			
Below 40%	7.9	36.3	32.6
40% to 80%	31.5	26.9	19.6
81% to 120%	24.4	17.7	13.7
Above 120%	36.2	10.6	11.7
Province of residence			
Newfoundland and Labrador	0.8	22.1	22.4
Prince Edward Island	0.4	20.9	15.9
Nova Scotia	2.8	23.6	21.0
New Brunswick	1.7	23.9	20.9
Quebec	30.0	15.7	10.3
Ontario	36.7	21.8	17.9
Manitoba	3.2	20.9	17.0
Saskatchewan	2.4	20.2	19.0
Alberta	8.8	18.8	20.8
British Columbia	13.0	20.2	19.9
Yukon	0.1	15.9	26.1
Northwest Territories	0.1	18.1	13.1
Nunavut	0.2	19.3	11.6
Dwelling satisfaction			
Very satisfied	23.1	15.6	10.6
Satisfied	47.7	16.9	14.0
Neither satisfied nor dissatisfied	18.3	23.3	22.6
Dissatisfied	8.5	30.0	27.0
Very dissatisfied	2.4	40.8	31.4
Dwelling issue			
No issue	64.2	16.2	12.9
One issue	25.1	23.5	19.8
Two issues	8.3	28.4	28.7
Three issues	2.5	33.8	26.6
Dwelling needs repair			
No	67.7	17.3	13.2
Yes	32.3	24.2	22.7
Residential mobility			
<2 years	25.3	15.8	17.2
2–5 years	33.6	16.9	15.6
6–9 years	19.7	20.4	16.8
10+ years	21.5	27.0	16.0
Neighbourhood satisfaction			
Very satisfied	33.4	14.6	11.8
Satisfied	44.1	20.1	15.6
Neither satisfied nor dissatisfied	12.9	25.4	24.5
Dissatisfied	5.1	29.4	27.4
Very dissatisfied	1.5	33.9	38.0
Neighbourhood safety			
Very safe	25.9	12.3	11.1
Reasonable safe	42.2	17.2	15.6
Somewhat unsafe	13.8	23.9	23.1
Very unsafe	3.6	34.6	30.6
Do not walk alone	14.5	31.1	17.8

Note: All percentages reported in this table are weighted and rounded.

**Table 3:** Association between tenancy housing type and self-reported general and mental health status.

Characteristics of Rental House	General Health Status (Poor)		Mental Health Status (Poor)	
	AOR ^†^ (95% CI)	*p*-Value	AOR ^†^ (95% CI)	*p*-Value
Tenancy housing type				
Cooperative	0.76 (0.70–0.83)	<0.001	0.58 (0.54–0.61)	<0.001
Not for profit	4.63 (4.15–5.16)	<0.001	1.26 (1.17–1.36)	<0.001
Government	0.67 (0.59–0.75)	<0.001	1.43 (1.34–1.52)	<0.001
Private	1.00		1.00	
Received Subsidy				
No	1.00		1.00	
Yes	1.21 (1.20–1.22)	<0.001	1.05 (1.03–1.06)	<0.001

^†^ Multivariable binary logistic regression model was adjusted with age, gender, ethnicity, education, occupation, family size, household composition, dwelling satisfaction, dwelling issue, dwelling needs repair, residential mobility, neighbourhood satisfaction, neighbourhood safety, household income ratio to province, province, and interaction terms. [Full regression model can be found in the Table S1].

## Data Availability

The data were obtained from the Research Data Centre (RDC) and are available with Statistics Canada’s permission.

## Supplementary Materials

The following supporting information can be downloaded at https://www.mdpi.com/article/10.3390/ijerph21091181/s1, Table S1. Characteristics of the tenants living in different types of rental housing, Canadian Housing Survey 2018–2019 (N = 26,371). Table S2. Full multivariable logistic regression model of factors associated with poor general health status of the tenants. Figure S1. Sensitivity analysis of the fitness model for general health (left) and mental health (right).
