# Supplementary material for: Rental Housing Type and Self-Reported General Health and Mental Health Status: Evidence from the Canadian Housing Survey 2018–2019
Source: Int J Environ Res Public Health. 2024 Sep 5;21(9):1181. doi: 10.3390/ijerph21091181 (PMC11431059; doi:10.3390/ijerph21091181)
Supplement: Supplementary file 1 [file ijerph-21-01181-s001.zip › ijerph-3128880-supplementary.pdf]

Table S1. Characteristics of the tenants living in different types of rental housing, Canadian Housing Survey 2018–2019.

Variables	Government (6.8%) Weighted %	Not-for-profit (3.1%) Weighted %	Co-operative (6.5%) Weighted %	Privately-owned (83.6%) Weighted %	Unweighted Sample size (N=26,371)
		Age (years)			
15-24	2.2	1.3	6.2	90.2	1,582
25-34	2.8	1.0	4.9	91.3	10,812
35-64	6.4	3.1	7.3	83.2	8,017
65+	11.6	5.9	7.7	74.8	5,960
		Gender			
Male	5.0	2.8	5.4	86.8	12,737
Female	7.9	3.4	8.1	80.6	13,634
		Ethnicity			
BIPOC	7.8	3.3	9.8	79.0	7,410
White	6.0	3.1	5.6	85.4	18,961
		Family size			
1	8.8	4.1	6.1	81.0	11,445
2	3.2	2.3	6.1	88.3	7,885
3	5.4	2.8	9.0	82.8	2,795
4	6.0	2.0	7.1	84.9	2,294
5+	8.6	2.8	9.6	79.0	1,951
		HH income category			
Below 40%	14.6	6.1	9.0	70.4	2,083
40% to 80%	11.8	5.0	8.8	74.4	8,307
80% to 120%	4.0	2.2	6.4	87.4	6,435
Above 120%	1.9	1.5	4.8	91.9	9,546

Note: All percentages reported in cells are weighted and rounded. Reported sample size counts are unweighted.

Table S2. Full multivariable logistic regression model of factors associated with poor general health status of the tenants.

Variables	General Health		Mental Health	
	OR (95% CI)	p-value	OR (95% CI)	p-value
	Tenancy type			
Government	0.67 (0.59-0.75)	0.000	1.43 (1.34-1.52)	0.000
Not for profit	4.63 (4.15-5.16)	0.000	1.26 (1.17-1.36)	0.000
Co-operative	0.76 (0.70-0.83)	0.000	0.58 (0.54-0.61)	0.000
Private	Ref.		Ref.	
	Received Subsidy			
No	Ref.		Ref.	
Yes	1.21 (1.20-1.22)	0.000	1.05 (1.03-1.06)	0.000
	Age (years)			
15-24	Ref.		Ref.	
25-34	1.60 (1.57-1.63)	0.000	0.94 (0.93-0.96)	0.000
35-64	2.64 (2.59-2.69)	0.000	0.81 (0.79-0.82)	0.000
65+	2.66 (2.60-2.72)	0.000	0.40 (0.39-0.41)	0.000
	Gender			
Male	Ref.		Ref.	
Female	0.83 (0.82-0.83)	0.000	1.12 (1.11-1.13)	0.000
	Ethnicity			
BIPOC	0.88 (0.87-0.89)	0.000	0.64 (0.64-0.65)	0.000
White	Ref.		Ref.	
	Education			
Less than high school	Ref.		Ref.	
High school diploma	0.81 (0.80-0.82)	0.000	0.91 (0.90-0.92)	0.000
Trade certificate/ diploma	0.73 (0.72-0.74)	0.000	0.83 (0.82-0.84)	0.000
College/non-university	0.78 (0.77-0.78)	0.000	0.81 (0.81-0.82)	0.000
University certificate/diploma below bachelor	0.64 (0.63-0.65)	0.000	1.06 (1.04-1.07)	0.000
Bachelor	0.57 (0.57-0.58)	0.000	0.87 (0.86-0.88)	0.000
University degree/diploma above bachelor	0.56 (0.55-0.57)	0.000	0.76 (0.75-0.77)	0.000
	Occupation			
Working	Ref.		Ref.	
Looking for work	1.17 (1.15-1.19)	0.000	1.58 (1.56-1.61)	0.000
Going to school	0.95 (0.93-0.96)	0.000	0.82 (0.81-0.83)	0.000
Keeping house	1.90 (1.87-1.93)	0.000	1.38 (1.35-1.41)	0.000
Caring for other family members including young children	1.62 (1.60-1.65)	0.000	1.25 (1.23-1.27)	0.000
Retired	1.78 (1.76-1.80)	0.000	0.98 (0.97-0.99)	0.005
Long term illness/disability	13.01 (12.86-13.15)	0.000	4.98 (4.92-5.03)	0.000
Doing volunteer work	1.30 (1.27-1.33)	0.000	1.51 (1.47-1.55)	0.000
No main activity	2.32 (2.28-2.35)	0.000	2.09 (2.06-2.13)	0.000
	Household or Family size			
1	Ref.		Ref.	
2	0.87 (0.86-0.88)	0.000	1.01 (0.99-1.02)	0.080
3	1.16 (1.14-1.18)	0.000	1.20 (1.89-1.22)	0.000
4	1.36 (1.33-1.38)	0.000	1.03 (1.02-1.05)	0.000
5+	1.24 (1.22-1.26)	0.000	0.97 (0.95-0.99)	0.002
	Household composition			
Couple with children	Ref.		Ref.	
Couple without children	1.48 (1.45-1.50)	0.000	1.70 (1.67-1.72)	0.000
Lone parent family	1.30 (1.28-1.32)	0.000	1.54 (1.52-1.56)	0.000

Person not in a census family	1.68 (1.65-1.70)	0.000	2.51 (2.47-2.55)	0.000
	Household income category			
Below 40%	Ref.		Ref.	
40% to 80%	1.26 (1.25-1.28)	0.000	1.04 (1.02-1.05)	0.000
80% to 120%	1.15 (1.13-1.17)	0.000	0.79 (0.77-0.80)	0.000
Above 120%	0.84 (0.83-0.85)	0.000	0.64 (0.63-0.65)	0.000
	Province			
Newfoundland and Labrador	Ref.		Ref.	
Prince Edward Island	1.04 (0.98-1.10)	0.169	0.69 (0.65-0.73)	0.000
Nova Scotia	1.17 (1.13-1.21)	0.000	0.79 (0.77-0.82)	0.000
New Brunswick	1.12 (1.08-1.16)	0.000	0.80 (0.77-0.83)	0.000
Quebec	0.64 (0.62-0.66)	0.000	0.35 (0.34-0.36)	0.000
Ontario	0.91 (0.88-0.93)	0.000	0.67 (0.65-0.69)	0.000
Manitoba	0.89 (0.86-0.92)	0.000	0.64 (0.62-0.67)	0.000
Saskatchewan	0.92 (0.89-0.95)	0.000	0.80 (0.77-0.83)	0.000
Alberta	0.94 (0.91-0.97)	0.000	0.86 (0.84-0.89)	0.000
British Columbia	1.05 (1.02-1.08)	0.001	0.79 (0.77-0.81)	0.000
Yukon	0.70 (0.64-0.77)	0.000	1.41 (1.31-1.51)	0.000
Northwest Territories	0.61 (0.54-0.68)	0.000	0.53 (0.74-0.59)	0.000
Nunavut	0.63 (0.59-0.68)	0.000	0.42 (0.38-0.46)	0.000
	Dwelling satisfaction			
Very satisfied	Ref.		Ref.	
Satisfied	1.09 (1.08-1.10)	0.000	1.18 (1.17-1.19)	0.000
Neither satisfied nor dissatisfied	1.62 (1.60-1.64)	0.000	1.80 (1.78-1.81)	0.000
Dissatisfied	1.88 (1.86-1.91)	0.000	1.85 (1.83-1.88)	0.000
Very dissatisfied	2.42 (2.38-2.47)	0.000	1.50 (1.47-1.53)	0.000
	Dwelling issue			
No issue	Ref.		Ref.	
One issue	1.35 (1.34-1.36)	0.000	1.23 (1.22-1.24)	0.000
Two issues	1.34 (1.33-1.36)	0.000	1.64 (1.63-1.66)	0.000
Three issues	1.53 (1.50-1.56)	0.000	1.24 (1.21-1.26)	0.000
	Household need repair			
No	Ref.		Ref.	
Yes	1.19 (1.18-1.19)	0.000	1.31 (1.30-1.32)	0.000
	Residential mobility			
<2 years	1.06 (1.05-1.07)	0.000	1.25 (1.24-1.27)	0.000
2 to <5 years	0.93 (0.92-0.94)	0.000	1.08 (1.07-1.08)	0.000
5 years to <10 years	0.85 (0.84-0.85)	0.000	1.02 (1.01-1.03)	0.000
10 years and more than 10 years	Ref.		Ref.	
	Neighbourhood satisfaction			
Very satisfied	Ref.		Ref.	
Satisfied	1.35 (1.34-1.36)	0.000	1.13 (1.12-1.14)	0.000
Neither satisfied nor dissatisfied	1.45 (1.44-1.47)	0.000	1.54 (1.52-1.55)	0.000
Dissatisfied	1.43 (1.41-1.45)	0.000	1.35 (1.33-1.37)	0.000
Very dissatisfied	0.90 (0.88-0.92)	0.000	1.47 (1.44-1.51)	0.000
	Neighbourhood safety			
Very safe	Ref.		Ref.	
Reasonable safe	1.37 (1.35-1.38)	0.000	1.26 (1.25-1.27)	0.000
Somewhat unsafe	1.62 (1.60-1.64)	0.000	1.52 (1.51-1.54)	0.000
Very unsafe	2.01 (1.98-2.04)	0.000	1.67 (1.64-1.70)	0.000
Do not walk alone	1.87 (1.85-1.88)	0.000	1.34 (1.32-1.35)	0.000
	Interactions:			
	Landlord type # Age			

Co-operative#25-34 years	1.27 (1.18-1.36)	0.000		
Co-operative#35-64 years	1.21 (1.13-1.30)	0.000		
Co-operative#65+ years	1.53 (1.43-.64)	0.000		
Not for profit#25-34 years	0.12 (0.11-0.13)	0.000		
Not for profit#35-64 years	0.22 (0.20-0.24)	0.000		
Not for profit#65+ years	0.17 (0.15-0.18)	0.000		
Government#25-34 years	1.32 (1.16-1.49)	0.000		
Government#35-64 years	2.19 (1.95-2.46)	0.000		
Government#65+ years	2.92 (2.60-3.29)	0.000		
Tenancy type # Received subsidy				
Co-operative#Yes			0.83 (0.80-0.85)	0.000
Not for profit#Yes			1.46 (1.40-1.52)	0.000
Government#Yes			1.41 (1.35-1.47)	0.000
Tenancy type # Household income				
Co-operative#40% to 80%	0.65 (0.63-0.68)	0.000	1.27 (1.22-1.31)	0.000
Co-operative#80% to 120%	0.62 (0.60-0.65)	0.000	0.86 (0.82-0.90)	0.000
Co-operative#Above 120%	0.41 (0.40-.043)	0.000	1.37 (1.32-1.44)	0.000
Not for profit#40% to 80%	0.75 (0.71-0.78)	0.000	0.94 (0.91-0.98)	0.005
Not for profit#80% to 120%	0.76 (0.72-0.81)	0.000	0.41 (0.39-0.44)	0.000
Not for profit#Above 120%	1.34 (1.27-1.42)	0.000	0.99 (0.93-1.05)	0.676
Government#40% to 80%	0.57 (0.56-0.59)	0.000	0.68 (0.66-0.70)	0.000
Government#80% to 120%	0.75 (0.72-0.78)	0.000	0.40 (0.38-0.041)	0.000
Government#Above 120%	0.77 (0.74-0.81)	0.000	0.86 (0.82-0.91)	0.000
Tenancy type # Ethnicity				
Co-operative#BIPOC	1.71 (1.67-1.75)	0.000	1.14 (1.12-1.17)	0.000
Not for profit#BIPOC	1.36 (1.31-1.41)	0.000	1.93 (1.85-2.00)	0.000
Government#BIPOC	1.49 (1.45-1.53)	0.000	1.37 (1.33-1.41)	0.000
Tenancy# Household composition				
Co-operative#Couple without children	1.41 (1.35-1.47)	0.000	1.68 (1.60-1.76)	0.000
Co-operative#Lone parent family	1.24 (1.19-1.29)	0.000	2.30 (2.20-2.39)	0.000
Co-operative#Person not in a census family	1.20 (1.15-1.24)	0.000	0.96 (0.92-1.00)	0.031
Not for profit#Couple without children	0.87 (0.82-0.93)	0.000	0.65 (0.60-0.70)	0.000
Not for profit#Lone parent family	0.88 (0.83-0.94)	0.000	2.22 (2.09-2.36)	0.000
Not for profit#Person not in a census family	1.60 (1.52-1.69)	0.000	1.23 (1.17-1.30)	0.000
Government#Couple without children	1.97 (1.88-2.06)	0.000	0.57 (0.53-0.60)	0.000
Government#Lone parent family	0.93 (0.90-0.97)	0.001	0.71 (0.68-0.74)	0.000
Government#Person not in a census family	0.90 (0.86-0.93)	0.000	0.54 (0.52-0.56)	0.000
Tenancy# Neighbourhood safety				
Co-operative#Reasonable safe			1.33 (1.28-1.38)	0.000
Co-operative#Somewhat unsafe			0.95 (0.91-0.99)	0.021
Co-operative#Very unsafe			1.29 (1.23-1.36)	0.000
Co-operative#Do not walk alone			1.17 (1.12-1.22)	0.000
Not for profit#Reasonable safe			0.66 (0.63-0.69)	0.000
Not for profit#Somewhat unsafe			0.34 (0.33-0.36)	0.000
Not for profit#Very unsafe			0.63 (0.59-0.68)	0.000
Not for profit#Do not walk alone			0.45 (0.43-0.48)	0.000
Government#Reasonable safe			0.94 (0.90-0.97)	0.000
Government#Somewhat unsafe			1.06 (1.01-1.10)	0.008
Government#Very unsafe			0.65 (0.62-0.69)	0.000
Government#Do not walk alone			1.16 (1.12-1.21)	0.000

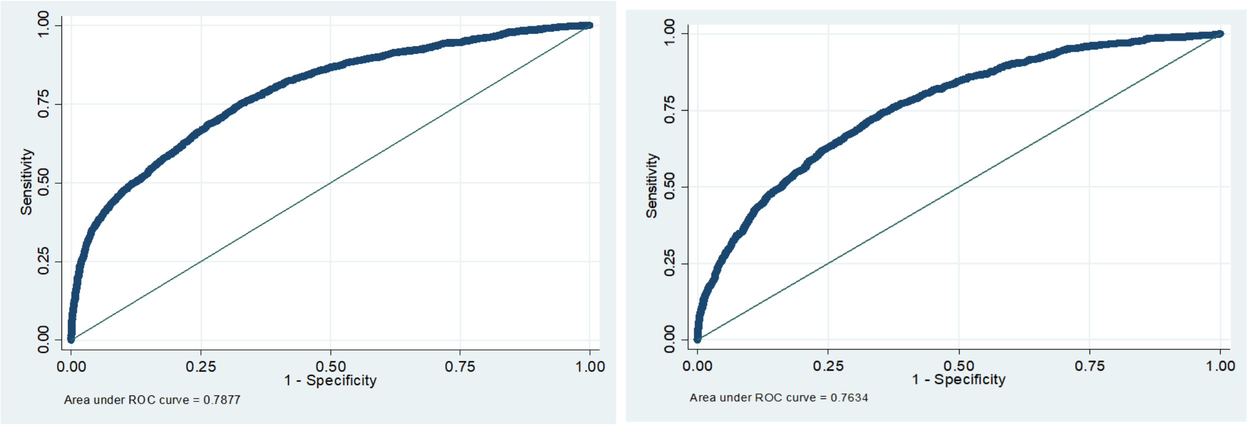

Figure S1. Sensitivity analysis of the fitness model for general health (left) and) mental health (right).
